# Helminthiasis: Hookworm Infection Remains a Public Health Problem in Dera District, South Gondar, Ethiopia

**DOI:** 10.1371/journal.pone.0144588

**Published:** 2015-12-10

**Authors:** Melashu Balew Shiferaw, Agmas Dessalegn Mengistu

**Affiliations:** 1 Bahir Dar Regional Health Research Laboratory Center, Bahir Dar, Ethiopia; 2 Felege Hiwot Referral Hospital, Bahir Dar, Ethiopia; UMASS Medical School, UNITED STATES

## Abstract

**Background:**

Intestinal parasitic infections are significant cause of morbidity and mortality in endemic countries. In Ethiopia, helminthiasis was the third leading cause of outpatient visits. Despite the health extension program was launched to address this problem, there is limited information on the burden of intestinal parasites after implementation of the program in our setting. Therefore, the aim of this study was to assess the intestinal helminthic infections among clients attending at Anbesame health center, South Gondar, Ethiopia.

**Methods:**

A cross sectional study was conducted at Anbesame health center from March to June 2015. A structured questionnaire was used to collect data from 464 study participants selected consecutively. Stool specimen collection, processing through formol-ether concentration technique and microscopic examination for presence of parasites were carried out. Data were entered, cleaned and analyzed using SPSS Version 20.

**Results:**

Among the total 464 study participants with median (±IQR) age of 25.0 (±21.75) years, 262 (56.5%) were females. Helminthic infection was found in 97 (20.9%) participants. Hookworm (68 [14.7%]) was the predominant parasite followed by *S*. *mansoni* (11 [2.4%]), *E*. *vermicularis* (9 [1.9%]) and *S*. *stercoralis* (5 [1.1%]). Patients with age group ≥15 years (AOR: 5.26; 95% CI: 2.05–13.46; P: 0.001) and walking barefoot (AOR: 2.20; 95% CI: 1.08–4.48; P: 0.031) were more vulnerable from the hookworm infections.

**Conclusions:**

There was a high burden of hookworm infections in our setting. Hence, regular shoes wearing, considering all age groups in the albendazole deworming as mass treatment and environmental hygiene are important interventions to reduce the burden of such neglected tropical disease.

## Introduction

Intestinal parasitic infections caused by intestinal helminths and protozoan are among the significant cause of morbidity and mortality in endemic countries. According to WHO estimate, 3.5 billion people were affected, and that 450 million were ill as a result of these infections [1, 2].

In sub-Saharan African countries where attributed largely to poor socioeconomic status, poor sanitation, inadequate medical care and absence of safe drinking water supplies; up to 250 million people are estimated to be infected with at least one or more species of intestinal nematodes [3]. In Ethiopia, helminthiasis was the third leading cause of outpatient visits [4]. Moreover, different studies showed higher prevalence of intestinal helminthiasis [5–7].

As a prevention strategy, Ethiopia launched the health extension program that promotes four areas of care: Disease prevention and control, family health, hygiene and environmental sanitation, and health education and communication [8]. This program is believed to reduce intestinal parasites burden if the environmental sanitation, and disease prevention and control is done as targeted. However, there is limited information on intestinal parasites burden after the program implementation. Therefore, the aim of this study was to assess the intestinal helminthic infections among clients attending at Anbesame health center, South Gondar, Ethiopia.

## Methods

A cross sectional study was conducted from March to June 2015 at Anbesame health center, Dera district, South Gondar. It is located at 11° 43^'^ 0^''^ North and 37° 38^'^ 0^''^ East. The climate of the area is Woinadega with 2077 meters above sea level. The area has 1300mm^3^ mean annual rain fall and 26°C mean annual temperature. The main activities of the people in this area are agriculture [9]. The health center serves for 45820 populations in the catchment area. Under this health center, five rural and one urban health posts are assigned to implement the health extension program. Health Extension Workers (HEWs) spend 75% of their time visiting families in their homes and performing outreach activities in the community. To address strong community demands for basic curative care, HEWs are trained to provide first aid; treat malaria, dysentery, intestinal parasites, and other ailments; and to refer cases to the nearest health center when more complicated care is needed [10]. 400 mg albendazole deworming has been given to <5 years children and to all pregnant women at 2^nd^ and 3^rd^ trimesters in the five health posts. The deworming coverage was 85.9% in these health posts [11].

A total of 464 clients following their health outcome at Anbesame health center were selected consecutively as study subjects. Clients treated for intestinal helminthiasis within one month were excluded from this study. Two trained interviewers collected the data from clients using a structured questionnaire containing socio-demographic variables, and prevention and control measures on intestinal parasites.

The laboratory investigation was performed by three laboratory technologists who have more than 5 years of experience in stool examination at health facilities in West Amhara region. About 2 grams of fresh stool specimen was collected from each study participant and placed in a labeled clean plastic stool container. Quality of reagents and instruments were checked before starting the laboratory investigation. A portion of stool was examined by direct wet mount with saline to observe motile intestinal parasites. The remaining stool specimen was processed by formol-ether concentration technique and examined under light microscope at 100X and 400X magnifications for the presence ova of parasites [12].

The data were entered, cleaned and analyzed using SPSS version 20. Frequencies of parasite burden were calculated. Multiple logistic regression was used to check associations with parasite burden. Variables having P value < 0.05 was taken as significant association.

This study was ethically reviewed and approved by the Amhara Regional Health Bureau Ethical Review Committee. Official permission was obtained from the Anbesame health center. Written informed consent, which was approved by the ethics committee, was obtained from each study participate. For participants under 18 years old, we obtained the written informed consent from their parents or guardians. Results were kept confidential and study participants found positive for intestinal parasites were linked to the health center's clinician.

## Results

### Socio-demographic characteristics

From the total of 464 study participants enrolled in this study, 262 (56.5%) were females. The median (±IQR) age of participants was 25.0 (±21.75) years. More than half of the participants were illiterate (284 [61.2%]) and married (257 [55.4%]). One hundred forty one (30.4%), 137 (29.5%) and 109 (23.5%) were farmers, house wives and students in occupation, respectively (Table 1).

**Table 1:** Sociodemographic characteristics of study participants at Anbesame HC, 2015.

Characteristics		Number	Percent
Age (years)	<15	132	28.4
	15–24	93	20.0
	25–49	184	39.7
	50+	55	11.9
Sex	Male	202	43.5
	Female	262	56.5
Marital status	Married	257	55.4
	Single	177	38.1
	Divorced	18	3.9
	Widowed	12	2.6
**Educational status**	College &above	13	2.8
	High school	32	6.9
	Elementary	135	29.1
	Illiterate	284	61.2
**Occupation**	Farmer	141	30.4
	Student	109	23.5
	Gov employee	13	2.8
	NA (under aged)	55	11.9
	Merchant	9	1.9
	House wife	137	29.5

### Intestinal parasites prevention and control practice

The participants got drinking water from river (289 [62.3%]), well (53 [11.4%]) and pipe (122 [26.3%]). Only 141 (30.4%) worn shoes regularly and 55 (11.9%) took 400 mg albendazole deworming. More than half (301 [64.9%]) of the participants had latrine. Of which, 242 (80.4%) did not use the latrine regularly. Most of the participants, 405 (87.3%), had open field defecation practice. Hand washing after toilet was not practiced in 374 (80.6%) clients.

### Helminthic infections

**Fig 1:**
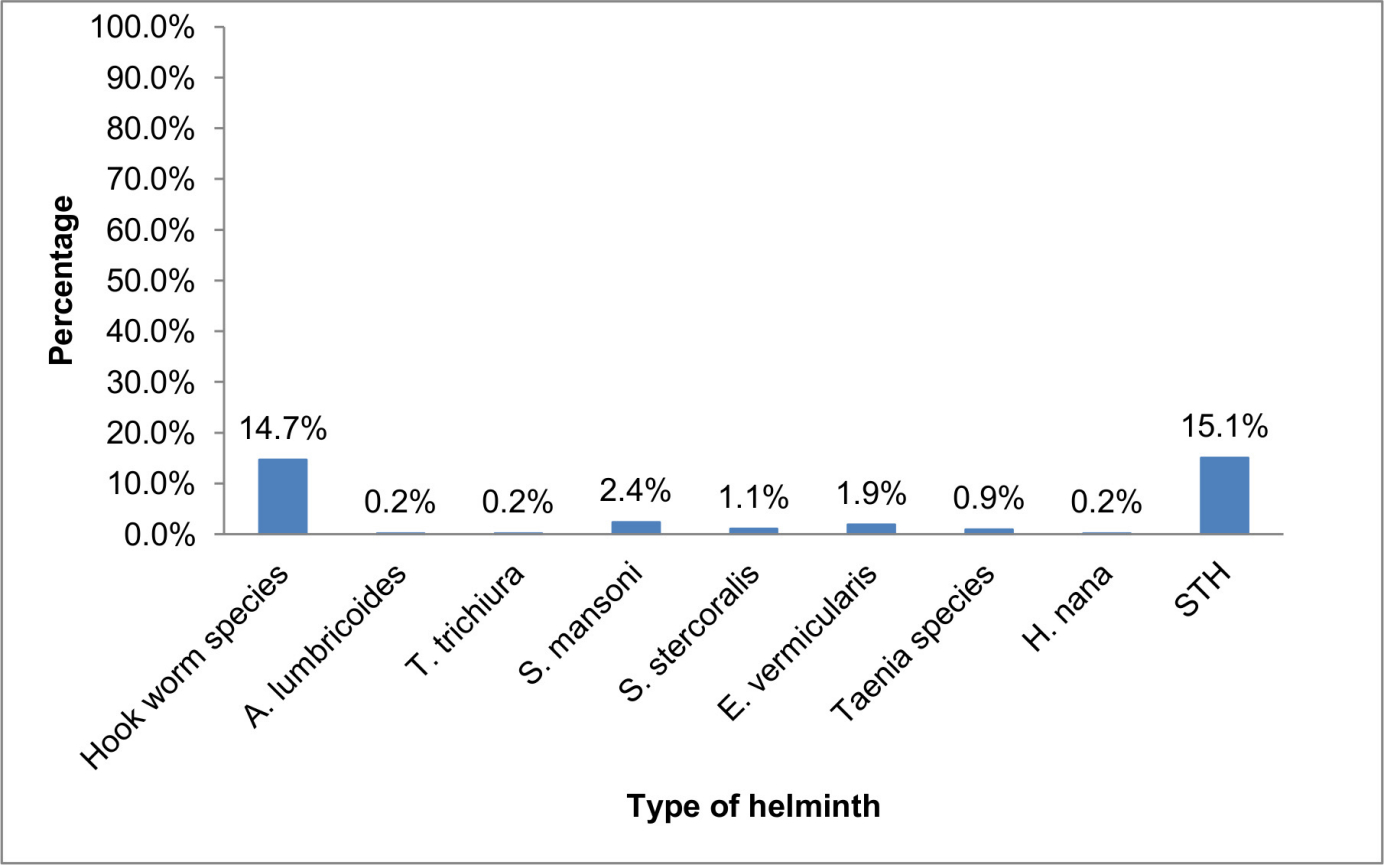
Proportion of helminthic infections among clients attending Anbesame health center, South Gondar, 2015. STH: Soil Transmitted Helminths

Of the total 464 stool specimens examined for intestinal parasites, the overall prevalence of helminthiasis was 20.9% (97/464). Soil transmitted helminths (hookworm, *A*. *lumbricoides* and *T*. *trichiura*) were found in 70 (15.1%) patients. Hookworm (68 [14.7%]) was the predominant parasite followed by *S*. *mansoni* (11 [2.4%]), *E*. *vermicularis* (9 [1.9%]) and *S*. *stercoralis* (5 [1.1%]) (Fig 1). Among the patients with hookworm infections, three patients were co-infected with *S*. *mansoni*, *E*. *vermicularis and S*. *stercoralis*.

### Determinant factors

In multivariate analysis, age group ≥15 years and walking barefoot were significantly associated with hookworm infections. Those clients with ≥15 years old had 5.26 (AOR: 5.26; 95% CI: 2.05–13.46; P: 0.001) times more to have hookworm infections (19.0%) compared to clients below 15 years (3.8%). The 17.6% of hookworm infections found among patients walking in barefoot was 2.21 (AOR: 2.21; 95% CI: 1.11–4.41; P: 0.024) times higher compared to those who worn shoes regularly (7.9%) (Table 2).

**Table 2:** Determinant factors for hookworm infection among clients attending at Anbesame health center, 2015.

Variables		Hookworm infection		COR (95% CI)	AOR (95% CI)	P
		No	Yes			
Ate unwashed fruits	No	113	14	1		
	Yes	283	54	1.54 (0.82–2.88)	-	-
Open field defecation	No	55	4	1		
	Yes	341	64	2.58 (0.90–7.37)	-	-
Walking barefoot	No	129	11	1	1	
	Yes	267	57	2.50 (1.27–4.94)	2.21 (1.11–4.41)	0.024
Took deworming/year	No	344	65	3.28 (0.99–10.80)	2.80 (0.83–9.40)	0.096
	Yes	52	3	1	1	
Age in year	<15	127	5	1	1	
	≥15	269	63	5.95 (2.34–15.15)	5.26 (2.05–13.46)	0.001

AOR: Adjusted Odds Ratio; CI: Confidence Interval; P: P value

## Discussion

Neglected disease including the three soil-transmitted helminths (ascariasis, hookworm infection, and trichuriasis) have been given relatively little attention by national governments and are considered to be low priority international public health issues [13]. However, these neglected diseases constitute major public health problems. For instance, hook worm infection can cause gastrointestinal blood loss, iron and energy deficiencies, protein and zinc deficiencies and thereby causing malnutrition and anemia [1, 14, 15]. In Ethiopia, helminthiasis was the third leading cause of outpatient visits [4].

Hygiene and environmental sanitation are the prevention strategies used in the health extension program that have been implemented through the seven packages (Proper and safe excreta disposal system; proper and safe solid and liquid waste management; water supply safety measures; food hygiene and safety measures; healthy home environment; arthropods and rodent control; personal hygiene) since 2005. HEWs spend 75% of their time visiting families in their homes and perform such activities in the community [8, 10]. However, this study has found that 20.9% prevalence of helminthic infections. Such high prevalence of helminthiasis is indicator of poor performance in the implementation of the prevention strategy.

Hookworm (14.7%) was the predominant parasite followed by *S*.*mansoni* (2.4%), *E*. *vermicularis* (1.9%) and *S*. *stercoralis* (1.1%). The prevalence may be much more especially for *S*.*mansoni* and *S*. *stercoralis* because of the less sensitive method (formal ether concentration technique) used to diagnose chronic strongyloidiasis in the stool of patient [16], and the only one day fecal examination used for *S*. *mansoni* diagnosis is not very sensitive.

In this study, 14.7% of the study participants were infected with hookworm. Similar prevalence of hookworm infections were reported: 11.5% in Delgi primary school [17], and 14.3% in Jimma [18]. However, the finding is higher compared to the 6.6% of hookworm prevalence reported at Teda health center [19], 4.9% in Northern Ethiopia [20] and 6.7% in Babile town, Eastern Ethiopia [21]. The possible reason might be due to improper latrine utilization, unavailability of safe drinking water, lower deworming coverage in the adolescents and adults and no hand washing practice after toilet since our finding has shown such problems.

The 17.6% of hookworm infections found among patients walking in barefoot was 2.21 (AOR: 2.21; 95% CI: 1.11–4.41; P: 0.024) times higher compared to those who worn shoes (7.9%). Similarly, studies conducted at Teda health Center, North Gondar [19] and in Zarima town, Northwest Ethiopia [7] reported significant association between walking barefoot and hookworm infections. This event happens when the infective stages of immature larva (filariform), which hatch in the soil, can penetrate human skin, and people usually become infected by walking barefoot on contaminated soil [22].

Moreover, clients with ≥15 years of age had significantly higher hookworm infections (19.0%) compared to those below 15 years (3.8%). This finding is supported by another study conducted in Southern Ethiopia [23]. This might be due to the difference in albendazole deworming that has been given to all <5 years children every 6 months and to all pregnant women at 2^nd^ and 3^rd^ trimester as a mass treatment in the HEP [11]. Our finding has also shown that significantly lower number of study participants above 15 years old took 400 mg albendazole deworming (9.6%) compared to the 17.4% of deworming utilization in under 15 years (P: 0.025).

This study was not without limitation. Due to budget constraint: Kato-Katz smear technique was not used, and only one day fecal examination was carried out to check the presence of helminths that could affect diagnostic sensitivity of parasites such us *S*. *mansoni*.

## Conclusions

There was a high burden of hookworm infections in our setting. Participants walking bare foot (AOR: 2.21; 95% CI: 1.11–4.41; P: 0.024) and age group ≥15 years (AOR: 5.26; 95% CI: 2.05–13.46; P: 0.001) were vulnerable from the hookworm infections. Hence, regular shoes wearing, considering all age groups in the albendazole deworming as mass treatment and environmental hygiene are important interventions to reduce the burden of such neglected tropical disease.
